# High-resolution peripheral quantitative CT of the proximal fifth metatarsal reveals microstructural similarity between Lawrence and Botte zones I and II

**DOI:** 10.1302/2046-3758.151.BJR-2025-0238.R1

**Published:** 2026-01-05

**Authors:** Lara Krüger, Ali Hedar, Tanja Spethmann, Benjamin Ondruschka, Frank Timo Beil, Felix von Brackel, Alexander Simon, Tim Rolvien

**Affiliations:** 1 Department of Trauma and Orthopaedic Surgery, Division of Orthopaedics, University Medical Center Hamburg-Eppendorf, Hamburg, Germany; 2 Institute of Anatomy and Experimental Morphology, University Medical Center Hamburg-Eppendorf, Hamburg, Germany; 3 Institute of Legal Medicine, University Medical Center Hamburg-Eppendorf, Hamburg, Germany; 4 Helios ENDO-Klinik Hamburg, Hamburg, Germany; 5 Department of Osteology and Biomechanics, University Medical Center Hamburg-Eppendorf, Hamburg, Germany

**Keywords:** Jones fracture, Metatarsal, foot, Microarchitecture, HR‐pQCT, fifth metatarsal, pQCT, bone mineral density, trabecular bone, regression analyses, volumetric bone mineral density, metatarsal fractures, BMI, diaphysis, stress fractures

## Abstract

**Aims:**

The optimal treatment of proximal fifth metatarsal (MT-V) fractures remains controversial. The most commonly used classification according to Lawrence and Botte distinguishes between three fracture types (zone I to III, from proximal to distal), aiming to derive the healing potential and treatment recommendations. It has been proposed that certain fracture types, such as zone II fractures, should be allocated to zone I and therefore treated conservatively rather than surgically based on their healing potential. Accordingly, this work aims to investigate the osseous microarchitecture in the three different zones of the proximal MT-V as a morphological basis for fracture classification.

**Methods:**

A total of 30 bilateral MT-V bones were obtained from 15 skeletally intact body donors. The zone-specific, trabecular, and cortical microarchitecture at the MT-V base was analyzed by high-resolution peripheral quantitative CT (HR-pQCT). Differences between zones I, II, and III were analyzed using group comparisons and the influence of possible demographic factors using linear regression analyses.

**Results:**

A decrease in trabecular bone microarchitecture with a simultaneous increase in cortical parameters was observed from zones I to III. Both trabecular and cortical parameters showed greater structural similarity between zones I and II than between zones II and III. Specifically, trabecular bone mineral density, area, and bone volume fraction, as well as cortical bone mineral density and cortical thickness, all exhibited smaller mean differences between zones I and II than between zones II and III. Female sex and advanced age were risk factors for poor microarchitecture in all three zones.

**Conclusion:**

Our observations support the assumption that, due to the closer microstructural similarity, zone II fractures might have a similarly good healing potential as zone I fractures. The observed deterioration of microarchitecture in women and the elderly might indicate an increased susceptibility to fracture in this patient group.

Cite this article: *Bone Joint Res* 2026;15(1):16–24.

## Article focus

- Analysis of zone-specific bone microarchitecture of the proximal fifth metatarsal using high-resolution peripheal quantitative CT (HR-pQCT).
- The possible influence of demographic factors such as age, sex, and BMI on the microstructure of the proximal fifth metatarsal.

## Key messages

- Lawrence and Botte zone II shows greater microstructural similarity to zone I than to zone III.
- Female sex and advanced age are risk factors for poor bone microarchitecture in all zones.

## Strengths and limitations

- This is the first study presenting the microarchitecture of the proximal fifth metatarsal.
- Due to the nature of body donation, the patients were of advanced age, which represents a possible limitation.

## Introduction

Metatarsal fractures are among the most common fractures of the foot and ankle, occurring at an incidence of approximately 15.6/100,000 people.^1^ Of these, the fifth metatarsal (MT-V) is most commonly affected at 68%.^2^ The majority (61% to 64%) of injuries to the MT-V are localized at the proximal end (i.e. the base) of the bone.^3,4^ Anatomically, various tendons and ligaments insert at the base of the MT-V, and a tight capsular-ligamentous unit braces it firmly to the cuboid and the fourth metatarsal bone. Due to the long lever of the diaphysis, this results in a high mechanical load at the transition between the metaphysis and diaphysis.^5-7^ In line with this, supination trauma is cited as the most common fracture mechanism,^3^ with direct trauma being less common.^2,7-9^ Additionally, an anterograde and retrograde vascular watershed with a correspondingly poor blood supply is present at this site.^5,10,11^ Restricted fracture healing with an increased risk of delayed union and pseudarthrosis has been described as a consequence.^10,11^

The most commonly used classification established by Lawrence and Botte^12^ distinguishes three anatomical zones of involvement: zone I proximal to the fourth to fifth metatarsal articulation (tuberosity avulsion fracture), which usually heals sufficiently under conservative treatment,^13-15^ zone II at the level of the articulation (metaphyseal-diaphyseal junction), where surgery is sometimes recommended,^16-19^ and zone III distal to the articulation (proximal diaphysis), which is preferably treated surgically.^16,17,20-22^ However, due to varying terminology, in particular the inconsistent use of the term “Jones fracture”, the literature is ambiguous regarding the optimal fracture therapy.^4,22-24^ Interestingly, there is particularly conflicting evidence whether zone II fractures have a similar favourable healing potential as zone I fractures and should be treated conservatively,^4,23,25,26^ or whether they have a similar poor healing potential as zone III fractures owing to the weak perfusion, and should instead be addressed surgically.^7,17,22^

The bone microarchitecture of the MT-V has not yet been investigated thoroughly, although it could provide a valuable contribution to the controversial debate regarding the optimal classification and treatment of proximal MT-V fractures. Likewise, the possible influence of demographic factors such as age, sex, and BMI on the microarchitecture of the MT-V has not yet been evaluated. Therefore, the aim of this study was to investigate the microarchitecture of the proximal MT-V with analysis of the specific subregions according to the three defined zones of MT-V fractures. In addition, the possible influence of various demographic factors was to be analyzed. The knowledge gained may provide important decision-making aids for the classification and treatment of patients with fractures of the proximal MT-V in daily practice.

## Methods

### Specimens

Overall, 30 whole bilateral MT-V bones were obtained from 15 skeletally intact body donors. Demographic characteristics of the study cohort are displayed in Supplementary Table i. Specimens were fresh-frozen at a temperature of -18°C immediately after preparation and thawed at room temperature before analysis. Postmortem records were reviewed to exclude individuals with conditions that might affect overall skeletal integrity (e.g., cancer, diabetes, glucocorticoid medication, or periods of immobilization) or local bone integrity (e.g., history of fracture or surgical reconstruction involving the foot). Each sample was examined by an experienced orthopaedic surgeon (LK) to exclude any visible pathology, including deformity, former fractures, and/or degenerative changes. Demographic data including sex, age, and BMI were recorded.

### High-resolution peripheral quantitative CT

**Fig. 1:**
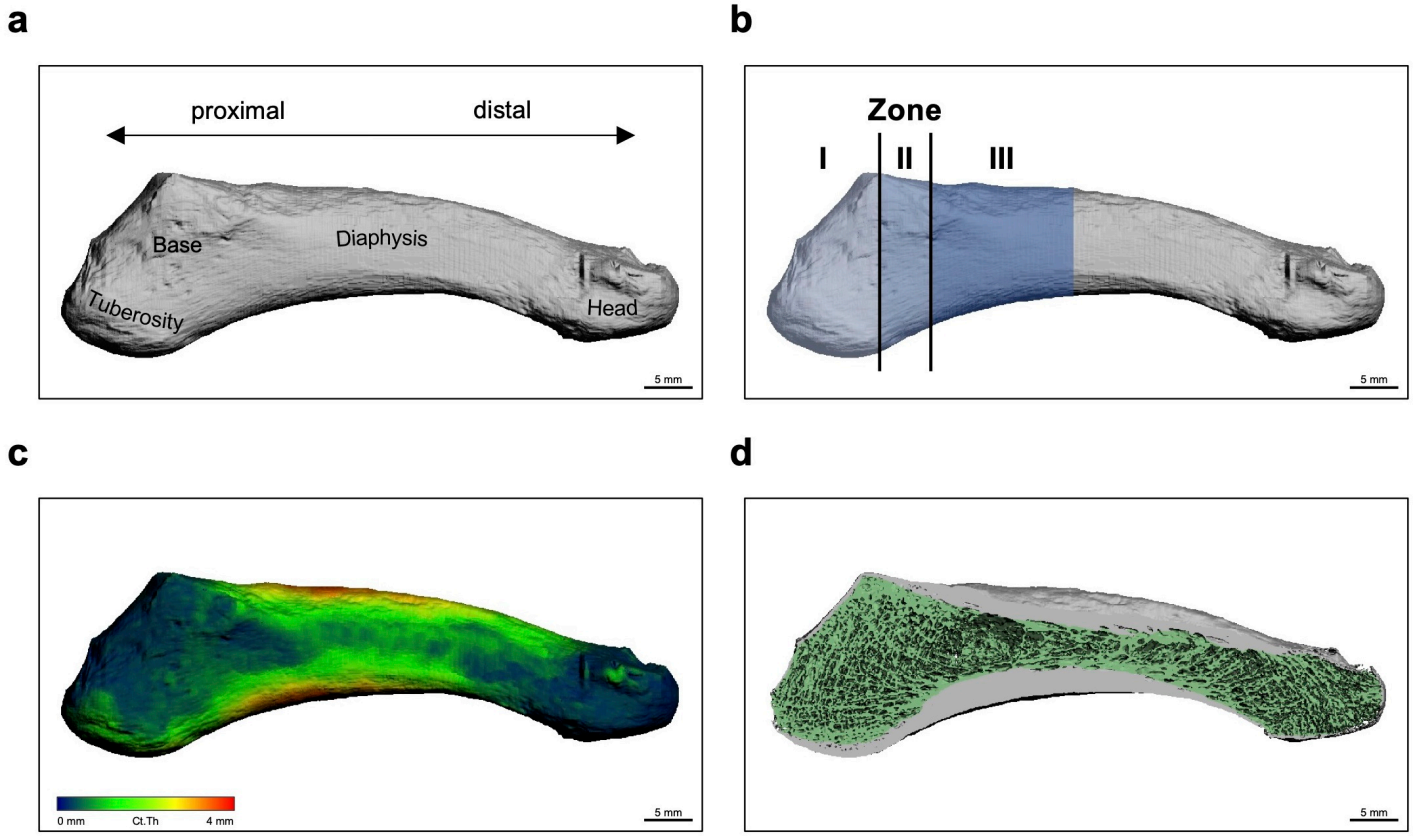
Anatomical overview of the fifth metatarsal bone (MT-V) based on high-resolution peripheral quantitative CT (HR-pQCT). a) External view and labelling of anatomical landmarks. b) Classification of the three zones (I to III) based on distinct anatomical landmarks, as described by Lawrence and Botte.^12^ Zone I extends from the most proximal aspect of the tuberosity of the MT-V to the beginning of the 4,5-intermetatarsal articulation (i.e., proximal to the 4,5-intermetatarsal articulation). Zone II extends from the end of zone I to the end of the 4,5-intermetatarsal articulation (i.e., at the height of the 4,5-intermetatarsal articulation). Zone III extends from the end of zone II (i.e., end of the 4,5-intermetatarsal articulation) and encompassed the proximal 1.5 cm of the MT-V diaphysis (i.e., distal to the 4,5-intermetatarsal articulation). c) Colour-coding of changes in cortical thickness. d) Representation of the cortical (grey) and trabecular (green) compartments.

HR‐pQCT measurements (XtremeCT II, Scanco Medical AG, Switzerland) of the MT-V were performed using the in vivo protocol (68 kVp, 1,470 µA, 200 ms integration time, voxel size of 30.3 µm) as described previously.^27,28^ A single measurement scan was performed to avoid stack shift. For optimal standardization, all scans were acquired by the same trained researcher (AS). All specimens were positioned with the same orientation and fixed within the manufacturer’s cast to prevent motion artifacts.^29^ For the analysis of specific subregions according to the Lawrence and Botte classification,^12^ three regions of interest were defined along the longitudinal axis of the MT-V based on distinct anatomical landmarks (Figures 1a and 1b). Zone I extends from the most proximal aspect of the tuberosity of the MT-V to the beginning of the 4,5-intermetatarsal articulation (i.e., proximal to the 4,5-intermetatarsal articulation, 391 ± 64 slices). Zone II extends from the end of zone I to the end of the 4,5-intermetatarsal articulation (i.e., at the height of the 4,5-intermetatarsal articulation, 338 ± 74 slices). Zone III extends from the end of zone II (i.e., end of the 4,5-intermetatarsal articulation) and encompassed the proximal 1.5 cm of the MT-V diaphysis (i.e., distal to the 4,5-intermetatarsal articulation, 500 slices each). This fixed-length region of zone III was selected in accordance with the original definition by Lawrence and Botte,^12^ who defined diaphyseal stress fractures as involving the proximal 1.5 cm of the shaft. A standard evaluation protocol provided by the manufacturer was used to generate 3D microarchitectural datasets of the cortical and trabecular compartments. The consistent quality of the scans was ensured by the daily use of the calibration phantom, and manual correction of the contours was performed if required. Volumetric bone mineral density (vBMD) including total vBMD (Tt.vBMD, mg HA/cm^3^), trabecular vBMD (Tb.vBMD, mg HA/cm^3^), and cortical vBMD (Ct.vBMD, mg HA/cm^3^) as well as bone microarchitecture parameters including bone volume to total volume (BV/TV, %), trabecular number (Tb.N, 1/mm), thickness (Tb.Th, mm), and separation (Tb.Sp, mm), cortical thickness (Ct.Th, mm), porosity (Ct.Po, %), and cortical pore diameter (Ct.Po.Dm, mm) were assessed according to current guidelines.^30^ Geometrical parameters including total area (Tt.Ar, mm^2^), trabecular area (Tb.Ar, mm^2^), cortical area (Ct.Ar, mm^2^), and cortical perimeter (Ct.Pm, mm^2^) were also analyzed.

### Statistical analysis

Statistical analysis was performed using GraphPad Prism 10.4.1 (GraphPad Software, USA). Normal distribution of the data was tested using the Shapiro-Wilk test. To test for the differences between three groups (zones I to III), one-way analysis of variance (ANOVA) with Tukey test was used for normally distributed data and Kruskal–Wallis *H* test with Dunn’s test for non-parametric data. The corresponding effect sizes were reported as η^2^ (0.02 $\overset{\wedge }{=}$ small, 0.13 $\overset{\wedge }{=}$ medium, 0.26 $\overset{\wedge }{=}$ large effect size) and Cohen’s d (0.2 $\overset{\wedge }{=}$ small, 0.5 $\overset{\wedge }{=}$ medium, 0.8 $\overset{\wedge }{=}$ large effect size). We also employed multiple linear regression models to assess the predictive value of sex (0 = female, 1 = male), age, BMI, and side (0 = left, 1 = right) on bone microarchitecture parameters in each of the three zones. For each model, all variables were entered simultaneously into the regression model (forced entry). Besides total model characteristics (adjusted R^2^ and p-value), individual regression coefﬁcients (standardized β), and p-values were calculated. Results are given as the mean (SD) and/or as boxplots showing the median (IQR) and individual data points. The level of significance was defined as p < 0.05 and exact p-values are reported unless p < 0.001.

## Results

### Subregional variations in microarchitecture

**Fig. 2:**
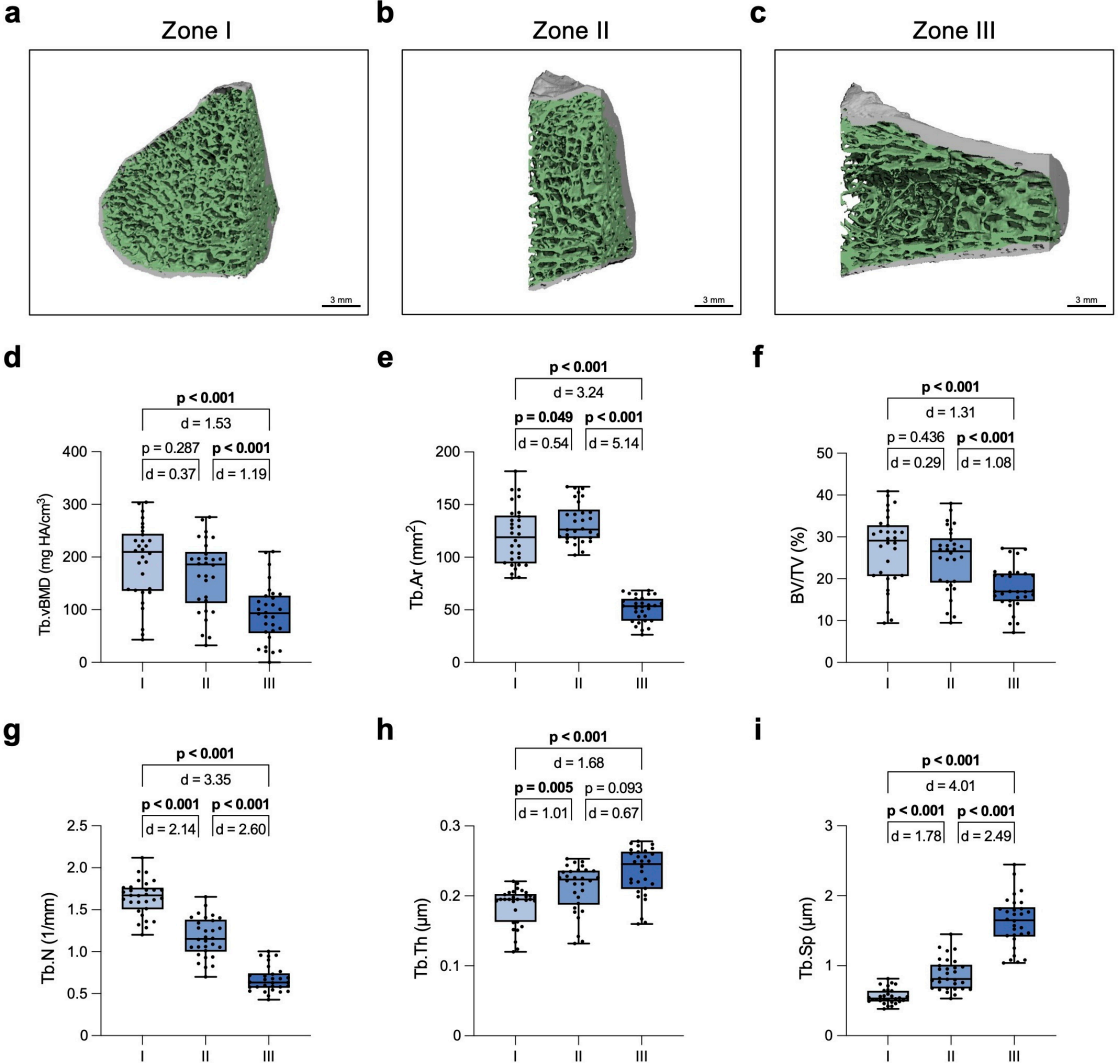
Assessment of the zone-specific trabecular proximal fifth metatarsal (MT-V) microarchitecture. Representative high-resolution peripheral quantitative CT (HR-pQCT) images of a) zone I, b) zone II, and c) zone III. The green colour-coding indicates the trabecular bone. Quantification and comparison of d) trabecular volumetric bone mineral density (Tb.vBMD), e) trabecular area (Tb.Ar), f) bone volume per tissue volume (BV/TV), g) trabecular number (Tb.N), h) trabecular thickness (Tb.Th), and i) trabecular separation (Tb.Sp). One-way analysis of variance (ANOVA) with Tukey test was used for normally distributed data and Kruskal–Wallis *H* test with Dunn’s test for non-parametric data. Exact p-values are reported unless p < 0.001.

Visual differences in the cortical and trabecular microarchitecture are shown for the entire MT-V bone in Figures 1c and 1d. Comparing densitometric, geometrical, and microstructural parameters of the trabecular compartment between the three zones (Figures 2a to 2c), we observed a general decrease in trabecular bone mass along zones I to III. However, we found that between zones II and III there was a strong difference in Tb.BMD, Tb.Ar, and BV/TV, while between zones I and II there was no difference or only a minor difference (Figures 2d to 2f). Interestingly, the overall decrease in trabecular microarchitecture was accompanied by a decrease in Tb.N and an increase in Tb.Sp, but also by an increase in Tb.Th, indicating that the trabeculae were thickest in zone III (Figures 2g to 2i).

**Fig. 3:**
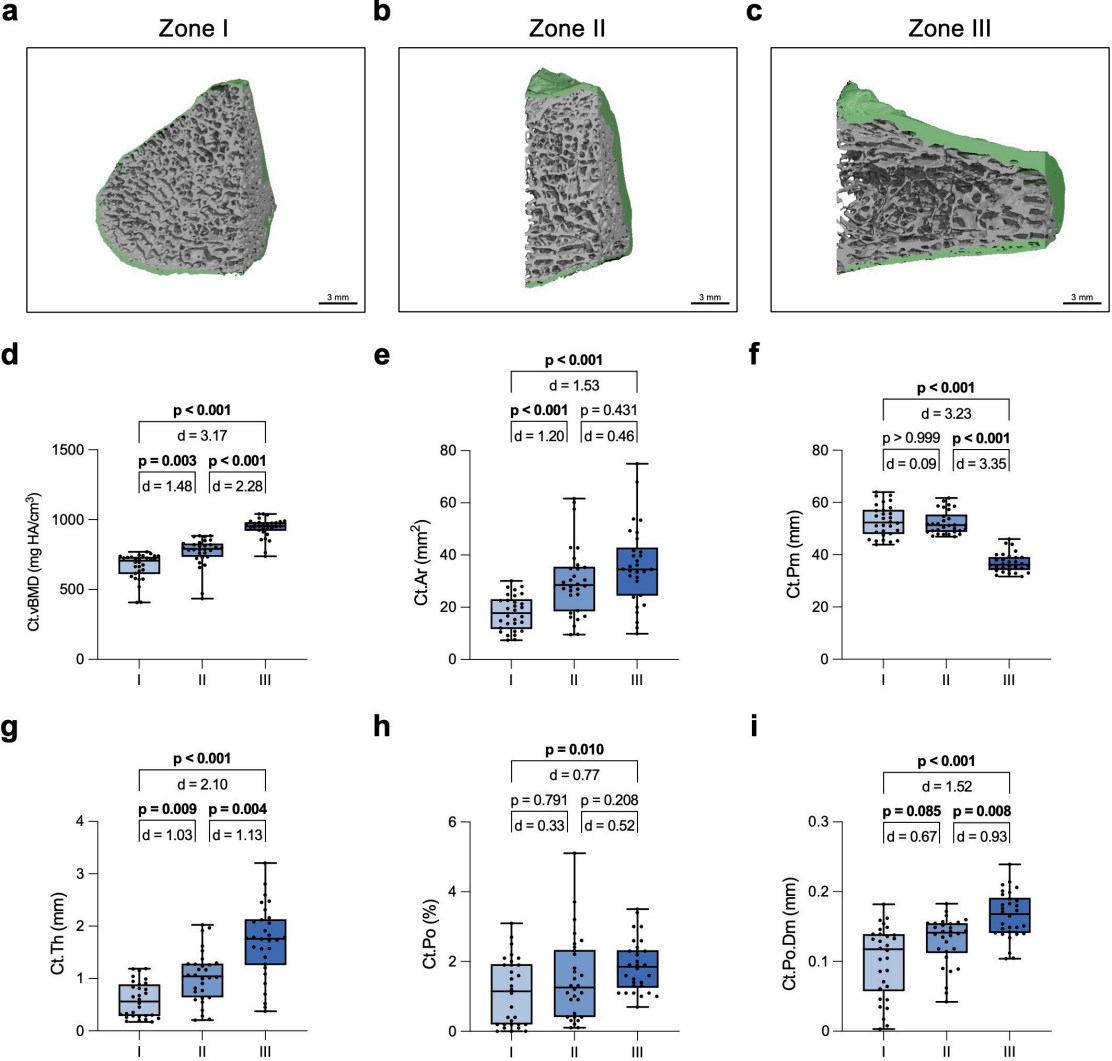
Assessment of the zone-specific cortical proximal fifth metatarsal (MT-V) microarchitecture. Representative high-resolution peripheral quantitative CT (HR-pQCT) images of a) zone I, b) zone II, and c) zone III. The green colour-coding indicates the cortical bone. Quantification and comparison of d) cortical volumetric bone mineral density (Ct.vBMD), e) cortical area (Ct.Ar), f) cortical perimeter (Ct.Pm), g) cortical thickness (Ct.Th), h) cortical porosity (Ct.Po), and i) cortical pore diameter (Ct.Po.Dm). One-way analysis of variance (ANOVA) with Tukey test was used for normally distributed data and Kruskal–Wallis *H* test with Dunn’s test for non-parametric data. Exact p-values are reported unless p < 0.001.

Assessment of subregion-specific cortical bone parameters (Figures 3a to 3c) showed, in contrast to the trabecular parameters, an increase in Ct.vBMD and Ct.Ar along the three zones (Figures 3d and 3e) and a decrease in Ct.Pm in zone III compared to zones I and II (Figure 3f). The evaluation of Ct.Th revealed that zones I and II as well as zones II and III differed significantly from each other, with a greater mean difference between zones II and III than between zones I and II, and the thickest Ct.Th observed in zone III (Figure 3g). The increase in Ct.Th was accompanied by an increase in Ct.Po and Ct.Po.Dm in zone III (Figures 3h and 3i). A summary of all trabecular and cortical HR-pQCT parameters of the MT-V subregions is displayed in Table I.

**Table I.** Overview of all mineralization, microarchitecture, and geometry results in the three zones assessed by high-resolution peripheral quantitative CT (HR-pQCT).

HR-pQCT parameter	Zone I (n = 30)		Zone II (n = 30)		Zone III (n = 30)		Total		I vs II		II vs III		I vs III	
	Mean	SD	Mean	SD	Mean	SD	p-value	η^2^	p-value	d	p-value	d	p-value	d
**Total**														
Tt.vBMD (mg HA/cm^3^)	254.0	90.6	276.7	98.9	443.2	159.6	< 0.001*	0.28	> 0.999	0.32	< 0.001	1.28	< 0.001	1.46
Tt.Ar (mm^2^)	136.7	29.8	161.4	26.1	85.8	16.3	< 0.001*	0.64	0.097	0.79	< 0.001	3.35	< 0.001	2.12
**Trabecular**														
Tb.vBMD (mg HA/cm^3^)	191.8	72.4	166.2	66.9	93.4	55.0	< 0.001†	0.30	0.287	0.37	< 0.001	1.19	< 0.001	1.53
Tb.Ar (mm^2^)	120.1	27.8	132.8	19.1	51.0	11.9	< 0.001†	0.76	0.049	0.54	< 0.001	5.14	< 0.001	3.24
BV/TV (%)	27.1	8.6	24.8	7.5	17.8	5.2	< 0.001†	0.24	0.436	0.29	< 0.001	1.08	< 0.001	1.31
Tb.N (mm^-1^)	1.645	0.210	1.169	0.236	0.674	0.154	< 0.001*	0.79	< 0.001	2.14	< 0.001	2.60	< 0.001	3.35
Tb.Th (mm)	0.185	0.027	0.211	0.034	0.234	0.035	< 0.001*	0.31	0.005	1.01	0.093	0.67	< 0.001	1.68
Tb.Sp (mm)	0.562	0.108	0.878	0.227	1.628	0.360	< 0.001†	0.76	< 0.001	1.78	< 0.001	2.49	< 0.001	4.01
**Cortical**														
Ct.vBMD (mg HA/cm^3^)	665.7	95.4	770.6	104.7	942.6	71.0	< 0.001*	0.69	0.003	1.48	< 0.001	2.28	< 0.001	3.17
Ct.Ar (mm^2^)	17.9	6.6	30.0	13.5	35.8	15.1	< 0.001*	0.30	< 0.001	1.20	0.431	0.46	< 0.001	1.53
Ct.Pm (mm)	52.9	5.9	52.3	4.4	37.0	3.7	< 0.001*	0.65	> 0.999	0.09	< 0.001	3.35	< 0.001	3.23
Ct.Th (mm)	0.594	0.339	1.035	0.489	1.708	0.691	< 0.001*	0.42	0.009	1.03	0.004	1.13	< 0.001	2.10
Ct. Po (%)	1.1	1.0	1.5	1.2	1.9	0.7	0.012*	0.08	0.791	0.33	0.208	0.52	0.010	0.77
Ct.Po.Dm (mm)	0.101	0.051	0.130	0.035	0.166	0.033	< 0.001*	0.29	0.085	0.67	0.008	0.93	< 0.001	1.52

* Kruskal–Wallis *H* test with Dunn’s test.

† One-way analysis of variance (ANOVA) with Tukey test.

BV/TV, bone volume to total volume; Ct.Ar, cortical area; Ct.Pm, cortical perimeter; Ct.Po, cortical porosity; Ct.Po.Dm, cortical pore diameter; Ct.Th, cortical thickness; Ct.vBMD, cortical volumetric bone mineral density; Tb.Ar, trabecular area; Tb.N, trabecular number; Tb.Sp, trabecular separation; Tb.Th, trabecular thickness; Tb.vBMD, trabecular volumetric bone mineral density; Tt.Ar, total area; Tt.vBMD, total volumetric bone mineral density.

### Demographic variables related to microarchitecture

Next, we analyzed whether microarchitectural parameters are related to demographic variables, including age, sex, BMI, and side (Table II). We found that sex was a robust independent determinant of almost all HR-pQCT parameters in all three zones, with women having poorer bone microarchitecture. We also demonstrated that age was negatively associated with Tt.vBMD, Tb.vBMD, BV/TV, Ct.Ar, and Ct.Th in all three zones. BMI did not appear to have an independent consistent effect on bone microarchitecture, with the exception of a significant negative effect on Tb.vBMD, BV/TV in zone I. In conclusion, female sex and higher age had a negative, subregion-independent effect on the microarchitecture of the MT-V, with a possible additional negative effect of obesity (high BMI) on the trabecular microarchitecture in zone I. The side, i.e. whether the right or left MT-V was examined, had no significant effect on any of the parameters recorded. Multiple linear regression models investigating the influence of sex, age, BMI, and side on all densitometric, geometrical, and microstructural HR-pQCT parameters are presented in Supplementary Tables ii to v.

**Table II.** Multiple linear regression model to determine the influence of sex, age, BMI, and side (left/right) on high-resolution peripheral quantitative CT parameters stratified by zones I to III.

Parameter	Zone I				Zone II				Zone III			
	Total model		β	p-value	Total model		β	p-value	Total model		β	p-value
**Tb.vBMD**												
Sex			0.571	0.002			0.461	0.007			0.503	0.010
Age	R^2^ adj. = 0.488		-0.613	< 0.001	R^2^ adj. = 0.523		-0.696	< 0.001	R^2^ adj. = 0.388		-0.488	0.003
BMI	p < 0.001		-0.398	0.026	p < 0.001		-0.324	0.057	p = 0.002		-0.104	0.575
Side			0.072	0.590			0.016	0.900			0.043	0.772
**Tb.Ar**												
Sex			-0.128	0.573			-0.155	0.445			-0.361	0.113
Age	R^2^ adj. = 0.041		-0.146	0.443	R^2^ adj. = 0.238		-0.127	0.457	R^2^ adj. = 0.080		0.190	0.310
BMI	p = 0.292		0.411	0.086	p = 0.028		0.617	0.006	p = 0.198		0.558	0.020
Side			-0.057	0.756			0.028	0.865			-0.038	0.835
**BV/TV**												
Sex			0.534	0.003			0.445	0.009			0.462	0.007
Age	R^2^ adj. = 0.492		-0.639	< 0.001	R^2^ adj. = 0.537		-0.711	< 0.001	R^2^ adj. = 0.522		-0.592	< 0.001
BMI	p < 0.001		-0.383	0.031	p < 0.001		-0.320	0.057	p < 0.001		-0.049	0.764
Side			0.068	0.610			0.031	0.807			-0.004	0.976
**Ct.vBMD**												
Sex			0.677	0.001			0.489	0.036			0.437	0.056
Age	R^2^ adj. = 0.339		-0.385	0.021	R^2^ adj. = 0.073		-0.220	0.245	R^2^ adj. = 0.098		-0.328	0.084
BMI	p = 0.006		-0.419	0.038	p = 0.213		-0.280	0.228	p = 0.162		-0.315	0.170
Side			0.037	0.809			-0.027	0.882			0.065	0.716
**Ct.Ar**												
Sex			0.585	< 0.001			0.352	0.041			0.472	0.007
Age	R^2^ adj. = 0.656		-0.549	< 0.001	R^2^ adj. = 0.491		-0.548	< 0.001	R^2^ adj. = 0.502		-0.529	< 0.001
BMI	p < 0.001		-0.014	0.918	p < 0.001		0.116	0.495	p < 0.001		0.012	0.941
Side			0.030	0.788			0.011	0.936			-0.026	0.845
**Ct.Th**												
Sex			0.569	0.003			0.464	0.013			0.494	0.006
Age	R^2^ adj. = 0.428		-0.487	0.003	R^2^ adj. = 0.425		-0.530	0.001	R^2^ adj. = 0.491		-0.574	< 0.001
BMI	p = 0.001		-0.156	0.389	p = 0.001		-0.066	0.714	p < 0.001		-0.102	0.548
Side			-0.005	0.969			0.031	0.828			-0.028	0.837

β represents the standardized regression coefficient.

BV/TV, bone volume to total volume; Ct.Ar, cortical area; Ct.Th, cortical thickness; Ct.vBMD, cortical volumetric bone mineral density; Tb.Ar, trabecular area; Tb.vBMD, trabecular volumetric bone mineral density.

## Discussion

It is accepted that the composition of a bone – in particular the relative proportions of trabecular and cortical structures – plays a crucial role in the healing process after fractures. In the case of proximal MT-V fractures, this is reflected in the different treatment concepts depending on which Lawrence and Botte zone is affected. However, the microarchitectural basis for this concept was not previously available. Despite the high incidence of MT-V fractures and the clinical relevance of possible complications such as delayed union and nonunion, there is still conflicting evidence regarding the optimal fracture treatment. Specifically, it has not been conclusively clarified whether zone II fractures show a comparable favourable healing potential to zone I fractures and should therefore be treated conservatively,^4,23,25^ or like zone III fractures and therefore addressed surgically.^7,17,22^ In the present study, we investigated the trabecular and cortical microarchitecture at the base of the MT-V bone according to zones I to III, and performed a regression analysis regarding possible demographic influencing factors. We observed a general decrease in trabecular bone mass along zones I to III. Interestingly, the trabecular parameters Tb.BMD, Tb.Ar, and BV/TV in particular showed a greater structural similarity between zones I and II, whereas pronounced differences were observed between zones II and III. Likewise, the cortical parameters, specifically Ct.BMD and Ct.Th, revealed a smaller mean difference between zones I and II than between zones II and III. Regarding the possible influence of demographic parameters, female sex and advanced age were robust risk factors for impaired microarchitecture. This observation was made in all three Lawrence and Botte zones. Notably, a high BMI was shown to be a possible additional negative influencing factor with poorer Tb.vBMD and BV/TV in zone I.

The observation that zones I and II are more alike than zones II and III with regard to trabecular and cortical parameters is an important contribution to the controversial discussion on the optimal treatment of zone II fractures. There are a considerable number of scientific reviews recommending surgical treatment for zone II fractures, particularly those with high functional demands, dislocation > 2 mm, and intra-articular or multifragmentary fractures.^7,18,21,22^ Chuckpaiwong et al^17^ even suggested combining zone II and III fractures as one entity, as treatment and outcome would be identical. However, a closer look into the primary studies on which the reviews are based reveals an inconsistent terminology, in particular the heterogeneous use of the term “Jones fracture” for zone II or zone III fractures.^20,31^ Other studies base their recommendation solely on the opinion of the authors, rather than actual evidence.^7,32^ In 2012, Polzer et al^26^ conducted a systematic review and, in contrast, described a good to excellent healing potential of zone II fractures with functional conservative therapy. As a consequence, they suggested combining zone I and II fractures as one entity due to their shared healing potential, and henceforth referring to them as “epimetaphyseal fractures”. Baumbach et al^23^ treated 39 patients with zone I and zone II fractures with functional conservative treatment and found full recovery in all patients, regardless of displacement, intra-articular involvement, or number of fracture fragments. Several other authors have likewise observed sufficient therapeutic outcomes with conservative treatment of zone II fractures.^33-36^ Our observations that the microarchitecture of Lawrence and Botte zone II more closely resembles that of zone I than zone III support the conclusion that zone II fractures may have a similar healing potential to zone I fractures.

Regarding the influence of demographic parameters, female sex and advanced age had a negative, zone-independent effect on the microarchitecture of the MT-V. This finding is consistent with other reports and similar observations in various other skeletal regions.^28,37,38^ Epidemiological studies have reported a higher proportion of both younger men and older women suffering from metatarsal fractures.^2,39^ At least the high incidence of fractures in older women can be explained by the known impairment of bone metabolism and osteoporosis due to age and sex.^40,41^ Interestingly, however, there is conflicting evidence regarding the relationship between osteoporosis and metatarsal fractures. While some studies have observed an increased risk of spinal osteoporosis in patients with metatarsal fractures,^42,43^ other studies found no association between foot fractures and reduced BMD.^8,44,45^ In particular, no correlation between BMD and fracture localization at the proximal MT-V could be determined in one study.^8^ It is well established that fracture resistance depends on trabecular and cortical geometry, mineralization, microarchitecture, and other bone quality factors.^28^ A recent meta-analysis found that HR-pQCT performed at reference sites (distal radius, tibia) can predict fracture incidence, with total and trabecular vBMD in particular having fracture predictive deficits^46^ The age- and sex-specific deterioration of microarchitecture that we observed in the MT-V base, including total and trabecular vBMD, suggests an osteoporosis-related fracture susceptibility of the MT-V base as well. Further studies are needed to clarify the relationship between low BMD and MT-V fractures.

Despite the application of a well-established method and the novelty of our data, some limitations need to be discussed. Due to the nature of body donation, only a patient cohort of rather advanced age was available. This may limit clinical implications for fracture patterns in younger patients who suffer mainly from highly traumatic and stress fractures. Furthermore, only fully intact MT-V bones were included, preventing the possibility of studying the microarchitecture of patients with former or subacute MT-V fractures.

In conclusion, we have provided novel data on the bone microarchitecture of the MT-V using HR-pQCT. We found a greater similarity between zones II and I than between zones II and III, providing evidence for considering zones I and II together as epimetaphyseal fractures. We also found an age- and sex-related deterioration in the microstructure of the MT-V (regardless of zone) which, in the context of existing evidence, suggests an osteoporosis-related fracture susceptibility of the MT-V base.

## Data Availability

The data that support the findings of this study are available from the corresponding author upon reasonable request.
